# Familial hemophagocytic lymphohistiocytosis type 5 in a Chinese Tibetan patient caused by a novel compound heterozygous mutation in STXBP2

**DOI:** 10.1097/MD.0000000000017674

**Published:** 2019-10-25

**Authors:** Xue Tang, Xia Guo, Qiang Li, Zhuo Huang

**Affiliations:** a Department of Pediatrics, West China Second University Hospital; b Key Laboratory of Birth Defects and Related Diseases of Women and Children, Sichuan University, Ministry of Education, Chengdu, Sichuan, China.

**Keywords:** diagnosis, familial hemophagocytic lymphohistiocytosis type 5 (FHL-5), mutation, *STXBP2*

## Abstract

**Rationale:**

Familial hemophagocytic lymphohistiocytosis (FHL) is a fatal autosomal recessive immunodeficiency disease whose rapid and accurate diagnosis is paramount for appropriate treatment. Mutations in *STXBP2* gene have been associated with FHL type 5 (FHL-5). Here, we report the first Tibetan Chinese patient diagnosed with FHL-5 caused by a novel compound heterozygous mutation in *STXBP2*.

**Patient concerns:**

A 9-year-old girl who presented with recurrent fever, splenomegaly, pancytopenia, hypofibrinogenemia, and conspicuous bone marrow hemophagocytosis was diagnosed with haemophagocytic lymphohistiocytosis (HLH).

**Diagnosis:**

FHL mutation analysis of the patient and her parents revealed that she presented compound heterozygosity for *STXBP2*: a novel missense mutation c.663G > C (p.Glu221Asp) and the known pathogenic splice-site mutation c.1247-1G > C (p.Val417LeufsX126). Bioinformatics analyses predicted that the new mutation was pathogenic and the FHL-5 diagnosis was confirmed.

**Interventions:**

Upon diagnosis, HLH-2004-directed chemotherapy was instituted, but there was a relapse. Allogeneic hematopoietic stem cell transplantation (HSCT) was performed.

**Outcomes:**

After transplantation, the patient presented implantation dysfunction, chronic graft-versus-host disease, and 5 episodes of pancreatitis. A follow-up after 5 years revealed that the patient had died of pancreatitis.

**Lessons:**

This finding expands the spectrum of FHL-5-related mutations in Chinese patients and indicates a clear genotype-phenotype correlation of FHL-5 in China.

## 1 Introduction

Familial hemophagocytic lymphohistiocytosis (FHL) is a genetically heterogeneous autosomal recessive immune disorder characterized by poor clearance of target cells and perpetual state of immune activation.^[1]^ The mutated genes in FHL lead to the deficiency of T lymphocyte and natural killer (NK) cell degranulation by inhibiting cytotoxic granule components or impairing their fusion machinery.^[2]^ It is estimated that the incidence of FHL is approximately 0.12 to 0.15 per 100,000 children per year and FHL-5, which is caused by mutations of *STXBP2,* only accounts for 10% of all FHL cases.^[2,3]^ Here we present a 9-year-old Tibetan Chinese patient with a novel compound heterozygous mutation in *STXBP2* who developed FHL-5 and died even after allogeneic hematopoietic stem cell transplantation (HSCT).

## 2 Case report

**Figure 1:**
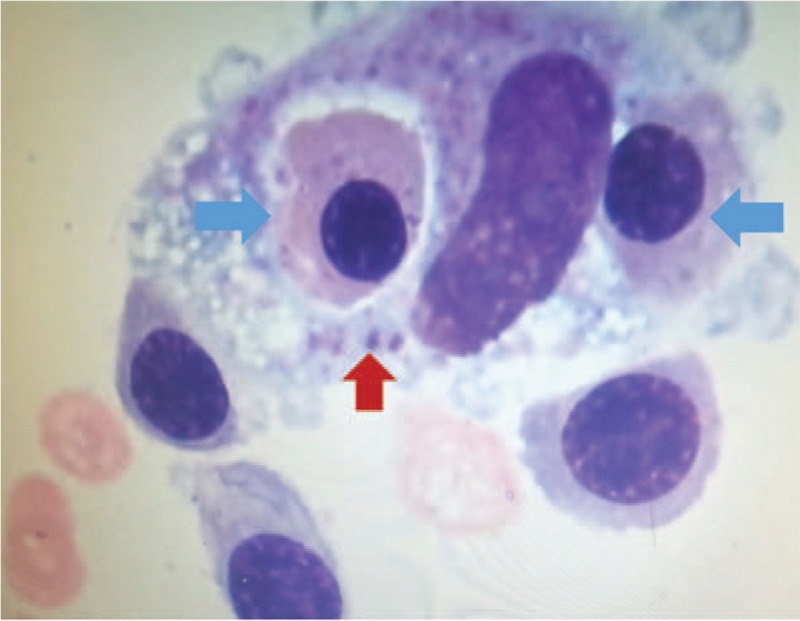
Phagocytosis was clearly observed in the bone marrow. The blue arrow indicated the phagocytosis of late erythrocyte and the red arrow showed platelet phagocytosis.

A 9-year-old girl was admitted to our hospital in 2013 with recurrent fever, splenomegaly, and pancytopenia that had lasted more than 1 month. She was a Chinese Tibetan girl in previous good health and had been living in Tibet, China since her birth. The patient was pallid and presented ecchymosis and edema of the lower extremities. The spleen was palpated 4 cm below the left costal margin. The patient's parents had never presented similar symptoms and she had no siblings. Complete blood count revealed that hemoglobin was 58 g/L, absolute reticulocyte count was 70 × 10^9^/L, white blood cells were 2.0 × 10^9^/L, absolute neutrophils were 0.22 × 10^9^/L, and platelets were 20 × 10^9^/L. Liver function indicated hypoproteinemia (albumin was 27.7 g/L). Serum ferritin was significantly elevated (1204.3 ng/ml) and coagulation screening tests suggested hypofibrinogenemia (145 mg/dl). Total plasma triglycerides and cerebrospinal fluid examination were normal. Bone marrow aspiration showed conspicuous hemophagocytosis and no malignant cells (Fig. 1). Though NK cell activity and sCD25 were not detected due to laboratory limitations, the diagnosis of hemophagocytic lymphohistiocytosis (HLH) was still made according to the diagnostic guideline criteria.^[4]^

**Figure 2:**
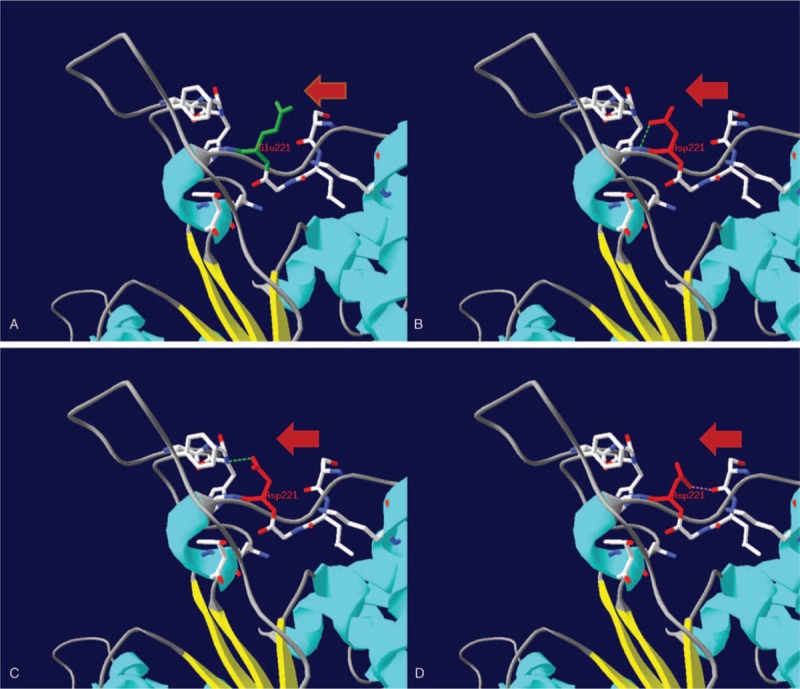
3D models of human Munc18-2 bearing wild-type (green) and mutant amino acid residues (red) of the novel missense mutation Glu221Asp. The hydrogen bonds (H-bonds) are shown by the green dotted line. The pink dotted line indicates steric clash between atoms. (A) Wide type. (B) Mutant model 1: new H-bond with Gly222. (C) Mutant model 2: new H-bond with His518. (D) Mutant model 3: steric clash with Ser218.

**Figure 3:**
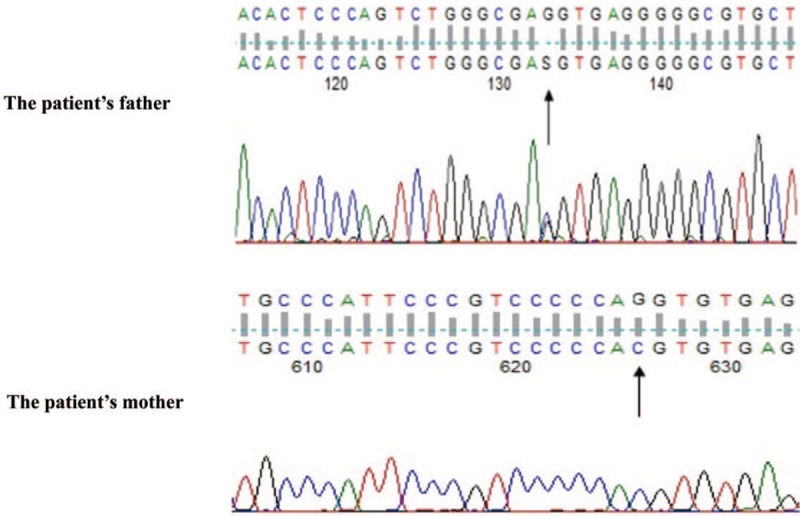
Sanger sequencing results in *STXBP2* of our patient's parents: her father was a heterozygote of c.663G > C and mother was a homozygote of c.1247-1G > C.

To elucidate the underlying etiology of HLH, series examinations were performed. Serological investigations for the presence of Epstein-Bar virus, rubella, cytomegalovirus (CMV), herpes simplex virus, hepatitis B, hepatitis C, human immunodeficiency virus (HIV), salmonella, and mycoplasma were all negative. There was no positive result in the autoimmune antibody tests. No mass was found on CT scans of head, chest, and abdomen. Finally, a genetic investigation was carried out by next-generation sequencing (NGS). The results revealed that the patient was a compound heterozygous in *STXBP2* for the following mutations: c.663G > C and c.1247-1G > C. The c.663G > C mutation in exon 8 was a novel missense variant (p. Glu221Asp) that is not listed in any known database. For better interpretation of the new variant, we adopted the recommendations of the American College of Medical Genetics and Genomics (ACMG) ^[5]^ and classified the novel mutation as likely pathogenic (PM1 + PM2 + PM3 + PP3 + PP4). To explore the potential structural changes induced by the new missense variant, a 3D structure of the mutant protein was generated with the Swiss-PdbViewer software using the crystallographic configuration (PDB id: 4CCA) of the human syntaxin binding protein 2 (also known as Munc18-2, encoded by *STXBP2*). The 3D mutant protein with p.Glu221Asp showed that the exchange to aspartic acid, which is smaller in size than the wild-type glutamic acid, might lead to the formation of a new hydrogen bond or a steric clash with neighboring residues (Fig. 2). The splice-site mutation in exon 15 (c.1247-1G > C) also results in an amino acid change (p. Val417LeufsX126) and has been reported as a pathogenic mutation.^[6]^ Moreover, genetic analysis of the parents DNA revealed that the father had the same heterozygous c.663G > C mutation (Fig. 3). Interestingly, the patient's mother had a homozygous c.1247-1G > C mutation and presented good health, even though she was 31 years old (Fig. 3). Therefore, the diagnosis of FHL-5 from 2 heterozygous mutations of *STXBP2* was unequivocally proven. Retrospectively, we confirmed the patient's parents had a non-consanguineous marriage.

HLH-2004-directed chemotherapy was instituted and the patient finally received a 29-week chemotherapy. Unfortunately, a relapse of HLH occurred soon after the treatment course, and the patient was submitted to haploidentical allogeneic HSCT, but presented implantation dysfunction, chronic graft-vs-host disease, and 5 episodes of post-transplant pancreatitis. On a follow-up after 5 years, the patient had died of pancreatitis and her mother had still not developed HLH.

## 3 Discussion

FHL-5 is associated with mutations in *STXBP2* and was initially identified as a genetic subtype of FHL in 2009 by zur Stadt et al^[6]^*STXBP2*, which has 19 exons, is located on 19p13 and encodes for the 593-amino acid Munc18-2 protein.^[1]^ Munc18-2 and syntaxin-11 (encoded by *STX11*) participate in T lymphocyte and NK cell cytotoxicity by regulating granule docking, initiating assembly and disassembly of the soluble N-ethylmaleimide-sensitive factor attachment protein receptor (SNARE) complex, and fusing lytic granules with the plasma membrane.^[7]^ Mutations in *STXBP2* can be identified throughout the entire coding region, including missense mutations, small deletions or insertions, and splice-site mutations.^[8]^ Of note, unlike other autosomal recessive genetic diseases, Spessott et al^[9]^ recently reported that the *STXBP2* R65 monoallelic mutation could contribute to FHL-5 development in a dominant-negative manner. In our patient, one novel missense mutation, c.663G > C (p.Glu221Asp), and 1 known pathogenic splice-site mutation (c.1247-1G > C)^[6]^ were detected by NGS in a compound heterozygous state of the *STXBP2* gene. Based on the visualization models for this novel mutation (Fig. 2), we hypothesize that the substitution of the conserved amino acid residue might affect protein folding, and/or reduce protein stability, causing its rapid degradation and finally resulting in decreased Munc18-2b expression. The classification of the novel missense mutation as likely pathogenic by ACMG standards ^[5]^ was performed in accordance with this assumption. Taken together, we infer that the novel missense mutation, along with the known pathogenic mutation, is responsible for the FHL-5 phenotype of this patient. Still, further in vitro biochemical studies are needed to confirm this hypothesis.

To date, FHL-5 has mainly been described in patients from the Middle East, of Turkish origin or European descent, who very often had consanguineous parents.^[6,10]^ Only limited data have been reported from Asian countries. To the best of our knowledge, this is the first case report of FHL-5 from a non-consanguineous family of Tibetan Chinese ethnicity. Distinct mutations may be associated with various ethnic origins and variable clinical presentations. Exon 15 splice-site mutations occur predominantly in German and Turkish patients and have not yet been reported in Asians.^[8]^ Patient age at diagnosis of FHL-5 with exon 15 splice-site mutations is significantly higher than that of patients with other mutations. Moreover, exon 15 FHL-5 shows a less severe NK cell and cytotoxic T lymphocyte (CTL) degranulation deficiency.^[8]^ The highest recorded onset age of an FHL-5 patient with exon 15 splice-site mutations is 19 years old.^[8]^ Our patient developed FHL-5 when she was 9 years old, thus qualifying as a late-onset FHL-5. Moreover, the levels of Munc18-2 and syntaxin-11 in patients with a homozygous missense mutation are markedly lower than those of patients with a homozygous splice-site mutation or with compound heterozygosity with a splice-site mutation.^[6]^ Therefore, we suppose this patient did not develop HLH until 9 years of age due to the compound heterozygosity of *STXBP2*, which includes the splice-site mutation in exon 15. Our case demonstrates that there is a clear genotype-phenotype correlation in FHL-5. Besides, in some late-onset FHL-5 patients, extracellular staining of CD107 and CTL cytotoxicity can reach the lower normal range.^[6]^ We therefore speculate that it is possible that the patient's mother's cytotoxicity and degranulation assays of NK cells and CTLs were in the lower normal range. Although the patient's mother has no current HLH-related clinical symptoms, we consider she has a high risk of developing HLH in the future with trigger factors such as infection, autoimmune disease, and tumors. However, there is a possibility that she may never develop HLH, and it would be very valuable to follow her up.

## 4 Conclusion

Early genetic testing is not only a rapid and accurate approach for the diagnosis of FHL-5, but it can also help in the identification of carriers and high-risk relatives who could receive genetic counseling. However, the mutation features of FHL-5 in China remain unclear. Our results expand the Chinese spectrum of pathogenic mutations in *STXBP2* and can definitely contribute to the elucidation of the genotype-phenotype correlation of FHL-5 in China.

## Acknowledgments

We thank the patient's family for their help and informed consent for publication of the case.

## Author contributions

**Conceptualization:** Xia Guo.

**Software:** Zhuo Huang.

**Supervision:** Xia Guo, Qiang Li.

**Writing - original draft:** Xue Tang.

**Writing - review & editing:** Xia Guo, Zhuo Huang.
